# Two Are Better than One: The Bi-Specific Antibody Mosunetuzumab Reveals an Improved Immune Response of Vγ9Vδ2 T Cells Targeting CD20 in Malignant B Cells in Comparison to the Mono-Specific Antibody Obinutuzumab

**DOI:** 10.3390/ijms26031262

**Published:** 2025-01-31

**Authors:** Lothar Marischen, Jürgen Fritsch, Jovana Ilic, Laura Wahl, Thomas Bertsch, Stefan Knop, Anna Bold

**Affiliations:** 1 Department of Hematology and Medical Oncology, Paracelsus Medical University, 90419 Nuremberg, Germany; 2 Department of Infection Prevention and Infectious Diseases, University Hospital of Regensburg, 93053 Regensburg, Germany; 3 Institute for Clinical Chemistry, Laboratory Medicine and Transfusion Medicine, Paracelsus Medical University, 90419 Nuremberg, Germany

**Keywords:** immunotherapy, γδ T cells, bi-specific antibodies, mosunetuzumab

## Abstract

In treating cancer, immunotherapy has been established as a later-line treatment option in clinical practice. That includes stem cell transplantation, modified or activated immune cells, and antibodies directed against aberrant cells. As an unconventional immune cell subgroup, γδ T cells have been shown to provide effects against malignant cells. They exhibit an MHC-independent activation process, which could diminish graft-versus-host disease after an adoptive transfer of allogeneic cells. Over the last years, the efficacy of therapeutic antibodies has been improved. As a bi-specific antibody, mosunetuzumab binds to both CD3 and CD20, thereby providing close proximity between effector and target cells. Here, we set out to analyze the efficiency of γδ T cells’ anti-tumor effects in combination with mosunetuzumab vs. the monoclonal anti-CD20 antibody obinutuzumab. Mosunetuzumab revealed improved responses of γδ T cells regarding their expression of IFN-γ and CD107a and their cytotoxicity towards malignant B cells from lymphoma B cell lines. In comparison to obinutuzumab, mosunetuzumab led to an equivalent or enhanced cytotoxicity against B cell lymphoma cell lines and primary patient samples, where this effect was even more prominent. In summary, we consider the combination of stimulated γδ T cells and mosunetuzumab to be a promising therapeutic approach for future clinical trials.

## 1. Introduction

While surgical treatment, radiotherapy, and chemotherapy are still indispensable for cancer treatment, immunotherapy has achieved vastly growing importance in that field. The transplantation of hematopoietic stem cells and the administration of genetically engineered, autologous chimeric antigen receptor (CAR) T cells, immune checkpoint inhibitors, antibody–drug conjugates, or monoclonal antibodies have increased treatment options rapidly. While the mono-specific anti-CD20 antibody rituximab revolutionized therapy of B cell lymphomas more than 20 years ago, similar antibodies with higher efficiency, such as obinutuzumab [1], were approved roughly 10 years ago. Even though obinutuzumab has improved efficacy over rituximab concerning the involvement of complement and/or cellular components of the immune system in the patient [2], the mode of action has not changed in general. While the binding of CD20 causes a direct activation of intracellular death-signaling pathways [3], it particularly marks the cell as a target for the patient’s immune system, as well. Various mono-specific CD20 antibodies, such as ofatumumab, rituximab, ublituximab, or obinutuzumab, bind to different distinct epitopes of the CD20 antigen. The details of this antibody/epitope interaction by different mono-specific CD20 antibodies are considered critical for their preclinical properties and clinical treatment efficiency [4]. Specifically, the interaction of obinutuzumab with the adequate binding site is analyzed in great detail, showing that obinutuzumab and rituximab recognize partly overlapping epitopes but bind with different orientations [5,6]. Mosunetuzumab and rituximab share the same anti-CD20 clone (2H7) and therefore the same epitope within CD20 [7,8,9]. Adding to that, obinutuzumab provides two binding sites for CD20, while mosunetuzumab exhibits only one per antibody. Consequently, the binding affinities of obinutuzumab and mosunetuzumab are understandably different [10]. The Fc region of the antibody interacts with appropriate receptors on immune cells of different kinds (for a review, please see [11]) and initiates immune reactions towards malignant B cells. On the other hand, a distinct group of bi-specific antibodies, commonly labeled “T cell engagers”, provides binding sites for both cancer and T cells. In addition to the activation of intracellular death-signaling pathways in B cells due to CD20 binding, mosunetuzumab brings targeted tumor and T cells in close proximity to enhance killing via perforin and granzyme release [12]. Even though bi-specific anti-CD20/anti-CD3 antibodies were established with conventional αβ T cells as the main effector cells in mind, an increasing amount of studies investigate γδ T cells as an emerging immunotherapeutic treatment option for cancer.

γδ T cells show a natural tropism for different tissues, including the mucosal epithelium and the tumor microenvironment, provide a battery of so-called natural killer receptors for targeting transformed cells, can be propagated ex vivo, and are activated independently of the major histocompatibility complex (MHC) [13,14]. The last feature, in particular, sets γδ T cells apart from αβ T cells by opening up an opportunity for cancer treatment through adoptive transfer of allogeneic immune cells without fulfilling critical MHC matching criteria. That, in turn, creates the possibility to generate high amounts of purified, stimulated, or potentially modified γδ T cells ex vivo for long-term storage before these cells are adoptively transferred to cancer patients at times and concentrations best for treatment. As an option for allogeneic cellular immunotherapy, γδ T cells may participate in fulfilling the promise of a cost-effective, scalable, and on-demand cancer treatment [15]. As a second advantage of MHC-independent activation, γδ T cells are not reliant on additional stimulation by dendritic cells but kill target cells directly. Vγ9Vδ2 T cells represent the main subpopulation of γδ T cells in human blood and can be activated by pyrophosphates overproduced by malignant cells. This initiates cytotoxic effects of Vγ9Vδ2 T cells, which produce tumor necrosis factor (TNF)-related apoptosis-inducing ligand (TRAIL), Fas-ligand (FasL), granzyme B, and perforin. In addition, Vγ9Vδ2 T cells produce interferon-γ (IFN-γ) to recruit other immune cells. This can be utilized for an in vitro or in vivo pharmaceutical stimulation of γδ T cells, as bisphosphonates, such as zoledronate, lead to an increased release of isopentenyl pyrophosphate by antigen-presenting cells [16]. As a first step towards the usage of γδ T cells in clinical cancer treatment, case studies with autologous [17], haploidentical [18], or allogeneic [19] γδ T cells were conducted. Phase 1 studies addressing the adoptive transfer of autologous [20,21] or allogeneic [22] γδ T cells revealed the feasibility, the safety, and, at least in part, the clinical success of this method. Currently, many clinical trials are carried out in parallel to characterize possible contributions to cancer treatment of autologous or allogeneic γδ T cells partly stimulated with zoledronate, interleukin-2 (IL-2), or vitamin C or D [23,24]. Their key features and encouraging results in clinical studies qualify γδ T cells as a promising off-the-shelf product for cancer treatment. With regard to a clinical approach for using a combination of therapeutic antibodies and γδ T cells, we already demonstrated that rituximab and obinutuzumab lead to enhanced antibody-dependent cellular cytotoxicity (ADCC) of γδ T cells in vitro [25]. In line with this, we recently used a combination of obinutuzumab and haploidentical γδ T cells in the individual treatment of a lymphoma patient [18].

Previous studies have discussed the benefits of γδ T cells and bi-specific antibodies in combination. For example, Oberg et al. showed the enhancement of γδ T cell cytotoxicity by an anti-Her2/anti-Vγ9 antibody against pancreatic cancer cells [26], Ganesan et al. described the impact of an anti-CD123/anti-Vγ9 antibody against acute myeloid leukemia cells [27], King et al. introduced an anti-EGFR/anti-Vδ2 antibody against Epidermal Growth Factor Receptor (EGFR)-expressing tumor cells [28], and Yang et al. used Vγ2Vδ2 T cells as effector cells against programmed cell death-ligand 1 (PD-L1)-expressing target cells through administration of an anti-PD-L1/anti-Vγ2 antibody [29]. In 2022, treatment with the bi-specific anti-CD20/anti-CD3 antibody mosunetuzumab was approved for use in the United States [30] and in the European Union [31]. With a substantially improved cultivation method for γδ T cells available [32], we further pursued the idea of immunotherapy with expanded allogeneic γδ T cells in combination with bi-specific antibodies within clinical framework conditions. Mosunetuzumab was chosen as representative for anti-CD20/anti-CD3 bi-specific antibodies for this study. For a comparative analysis, we opted for the mono-specific antibody obinutuzumab, as it binds to CD20, as well, outperformed rituximab in a clinical trial [2], led to an increased ADCC by γδ T cells in a number of CD20^+^ B cell lines [25], and was used in the above-mentioned individual treatment of a lymphoma patient [18]. In this preclinical study, we analyzed the benefits of using mosunetuzumab instead of obinutuzumab as a therapeutic antibody for enhancing the anti-tumor effects of γδ T cells.

## 2. Results

### 2.1. Effective In Vitro Concentration of Mosunetuzumab

**Figure 1:**
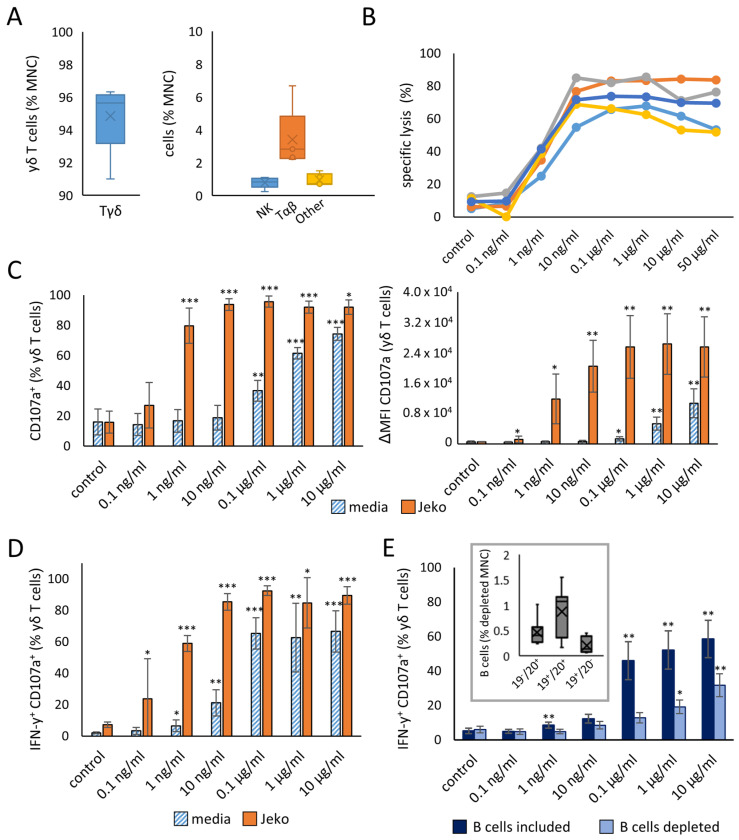
Effective in vitro concentration of mosunetuzumab. (**A**) Composition of effector cells generated through the isolation of MNCs of healthy donors and their stimulation with Zol/IL-2 for 9 days. (**B**) Effector cells were incubated with Jeko and mosunetuzumab in the indicated concentrations or with 1 µg/mL of non-specific isotype control IgG in an effector to target ratio of 6.7:1. After 4 h, target cell lysis was measured through flow cytometry. The different lines show the killing with the stimulated yδ cells of the different donors. (**C**) MNCs of healthy donors were isolated and stimulated with Zol/IL-2. After 11 days, effector cells were incubated with media control or Jeko and mosunetuzumab in the indicated concentrations or with 1 µg/mL of non-specific isotype control IgG. After 3 h, CD107a^+^ yδ T cells and Δmean fluorescence intensity (MFI) of CD107a on yδ T cells were detected through flow cytometry. (**D**) Freshly isolated, unstimulated MNCs were co-incubated with Jeko or media in the presence of different mosunetuzumab concentrations or 1 µg/mL of non-specific IgG control. After 48 h, the relative amount of yδ T cells expressing cytoplasmic IFN-y and surface CD107a was analyzed through flow cytometry. (**E**) B cells were depleted or not from freshly isolated MNCs, including yδ T cells. The diagram in the gray box shows the percentage of residual B cells in depleted MNCs. Undepleted or depleted MNCs were incubated for 48 h with IgG as the control or increasing concentrations of mosunetuzumab, as indicated. The relative amount of yδ T cells expressing cytoplasmic IFN-y and surface CD107a was analyzed through flow cytometry. The data are presented as mean ± standard deviation (SD) of 5 (**A**,**C**,**D**) or 8 (**E**) independent experiments. * *p* < 0.05, ** *p* < 0.01, *** *p* < 0.001 comparing mosunetuzumab results with the respective IgG control.

The bi-specific antibody mosunetuzumab is intravenously administered at a dose of 1 mg/mL into patients and reaches an average maximal serum concentration of about 17.9 µg/mL [33]. Accordingly, 50 µg/mL was considered an appropriate maximum concentration of mosunetuzumab in a dilution series to determine the effective concentration for in vitro experiments. To prove the specific target cell lysis of γδ T cells, which is considered the key criterion, mononuclear cells (MNCs) of healthy donors were isolated and stimulated with zoledronate/interleukin-2 (Zol/IL-2), resulting in a cell product consisting of more than 90% γδ T cells. Details of the composition of the stimulated cells are shown in Figure 1A. Specific target cell lysis was checked after the incubation of different mosunetuzumab concentrations from 0.1 ng/mL upwards with co-cultures of the stimulated γδ T cells and malignant B cells. As shown in Figure 1B, with 10 ng/mL of mosunetuzumab, substantial specific lysis of Jeko cells could be achieved. The expression of CD107a as a marker for the degranulation of stimulated γδ T cells increased with 0.1 μg/mL and 0.1 ng/mL of mosunetuzumab in the absence and presence of target Jeko cells, respectively (Figure 1C). Next, we investigated the effect of mosunetuzumab on the cytoplasmic IFN-γ as a marker for the activation of γδ T cells within freshly isolated, unstimulated MNCs. This is in contrast to Figure 1C, in which we analyzed the effect of mosunetuzumab on γδ T cells after stimulation with Zol/IL-2. After 48 h of co-culture with Jeko cells and the antibody, 0.1 ng/mL of mosunetuzumab proved to be sufficient for a significant effect on the relative amount of CD107a^+^/IFN-γ^+^ γδ T cells (Figure 1D). Interestingly, in the absence of malignant B cells, mosunetuzumab at a concentration of 1 ng/mL or higher revealed an effect on both CD107a and IFN-γ expression. This could be explained by a direct effect of mosunetuzumab on γδ T cells by binding via CD3/anti-CD3 or, alternatively, by still residual B cells, which were initially abundant in the MNCs. To further elucidate the latter possibility, we reduced the B cell population from freshly isolated MNCs (including γδ T cells) of healthy donors from 8.5% (±2.0%) to less than 2% on average or left it unchanged (Figure 1E, gray box). The expression of IFN-γ and CD107a by γδ T cells was measured after mosunetuzumab was added at different concentrations (Figure 1E). In CD19-depleted MNCs, the percentage of IFN-γ^+^/CD107a^+^ γδ T cells was slightly but still increased depending on the mosunetuzumab concentration after 48 h of co-culture. According to that data, a stimulation of γδ T cells just through the binding of mosunetuzumab should be regarded as a possible option. In MNCs containing CD19^+^ B cells, however, the percentage of IFN-γ^+^/CD107a^+^ γδ T cells was strongly increased by 0.1 µg/mL compared to 10 ng/mL of mosunetuzumab. Taken together, binding via anti-CD3/CD3 and stimulation by still residual B cells both seemed to participate in the enhancement of γδ T cell activation. Because the isotype control (IgG) and obinutuzumab concentration in our former studies [25,32] were always 1 µg/mL and 1 µg/mL of mosunetuzumab yielded distinct results, subsequent experiments were performed with 1 µg/mL until specifically indicated otherwise.

### 2.2. Efficiency Improvement Using Bi-Specific Mosunetuzumab vs. Mono-Specific Obinutuzumab

**Figure 2:**
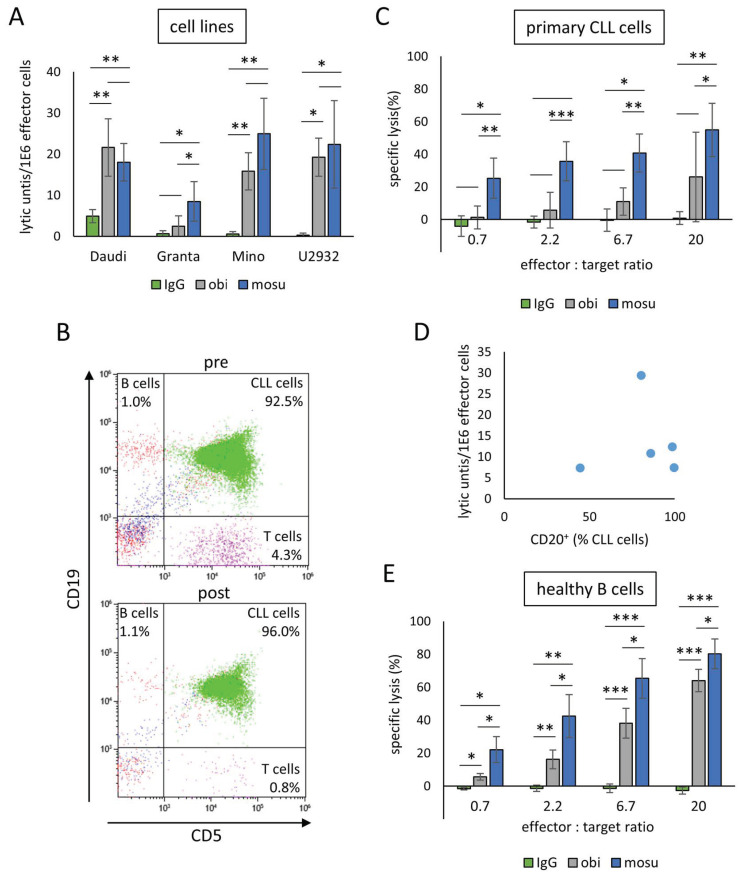
Efficiency improvement using bi-specific mosunetuzumab vs. mono-specific obinutuzumab. MNCs of healthy donors were isolated and stimulated with Zol/IL-2 up to 10 days, resulting in effector cells consisting of at least 90% yδ T cells. (**A**) At day 9 or 10 of cultivation, effector cells were incubated with different B cell lymphoma cell lines and 1 µg/mL of obinutuzumab (obi), mosunetuzumab (mosu), or non-specific isotype control (IgG) for 4 h. Lytic units per 1E6 effector cells were calculated based on the specific target cells’ lysis obtained with different effector to target ratios from 0.7:1 to 20:1. (**B**) MNCs from untreated CLL patients were isolated and depleted for CD3. Exemplary gating of CLL cells, B cells, and T cells pre- and post-CD3 depletion is shown. (**C**) At day 10 of cultivation, effector cells were incubated with primary CD3-depleted MNCs from CLL patients and 1 µg/mL of obinutuzumab (obi), mosunetuzumab (mosu), or non-specific isotype control IgG. After 4 h, specific lysis was measured through flow cytometry. (**D**) No correlation was found between the percentage of CD20^+^ CLL cells and the lytic units per 1E6 effector cells calculated based on the specific lysis from Figure 2C. (**E**) At day 8 of cultivation, effector cells were incubated with isolated B cells from healthy donors and 1 µg/mL of obinutuzumab (obi), mosunetuzumab (mosu), or non-specific isotype control (IgG) to perform a cytotoxicity assay. After 4 h, target cell lysis was measured through flow cytometry. The data are presented as mean ± SD of 5 (**A**,**C**,**D**) or 4 (**E**) independent experiments. * *p* < 0.05, ** *p* < 0.01, *** *p* < 0.001 comparing the results with the respective IgG control.

After determining an appropriate concentration of mosunetuzumab for in vitro experiments, a first comparative analysis of the effects of mosunetuzumab vs. obinutuzumab was carried out by adding these antibodies to a co-incubation of B cell lymphoma cell lines with stimulated γδ T cells while the ratio of effector (γδ T cells) to target (B cells) cells (E:T) was increased in a stepwise approach. For a concise presentation, the lytic units per 1E6 effector cells have been calculated based on the specific lyses obtained from all effector to target ratios. This revealed higher values for both therapeutic antibodies compared to the isotype control regardless of which B cell lymphoma cell line was used (Figure 2A). A significantly higher lysis for Granta, but not for Daudi, Mino, or U2932 could be observed with mosunetuzumab vs. obinutuzumab. To mimic a clinical scenario, primary cells from untreated chronic lymphocytic leukemia (CLL) patients were used as target cells. To eliminate unwanted effects of autologous CD3^+^ cells when co-culturing the malignant cells with mosunetuzumab and allogeneic Zol/IL-2-stimulated γδ T cells from a healthy donor, patients’ MNCs were depleted of CD3^+^ cells. After depletion, the target cells consisted, on average, of 94.6% (±1.5%) malignant cells, 0.7% (±0.3%) healthy B cells, and 0.8% (±0.3%) T cells, as shown in Figure 2B. When these cells were co-cultured with stimulated γδ T cells and respective antibodies, mosunetuzumab revealed much stronger lytic activity compared to obinutuzumab or an isotype control antibody, even at the lowest E:T of 0.7:1 (Figure 2C). Due to the simultaneous binding of CD20 and CD3 by mosunetuzumab, a possible correlation between CD20 expression levels of CLL-patient-derived B cells and the achieved lytic units was investigated and could be excluded (Figure 2D). Next, both antibodies and an IgG control were administered to co-cultures of γδ T and B cells of healthy donors. Mosunetuzumab led to significantly stronger specific lysis than obinutuzumab or the control antibody independently of the E:T considered (Figure 2E).

### 2.3. Mosunetuzumab Provides an Improved Killing Process

**Figure 3:**
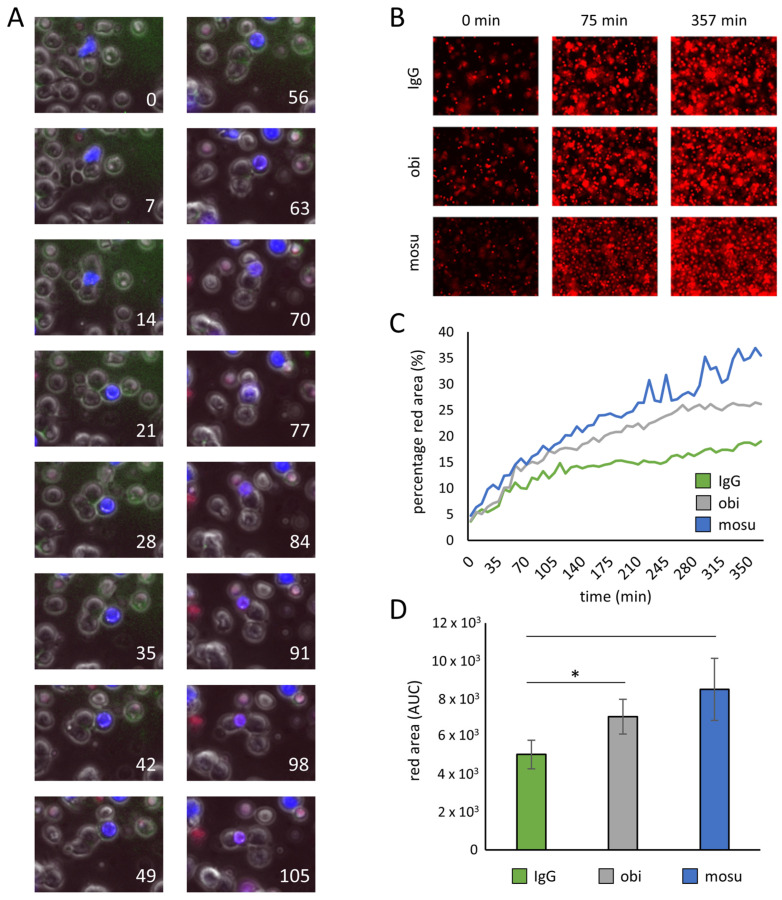
Live imaging of B and γδ T cell co-cultures w/o therapeutic antibodies through microscopy. B cell lymphoma cells were stained with Hoechst 33258 and incubated with Zol/IL-2-stimulated γδ T cells in a 1:2 ratio. Apotracker™ Green and 7AAD were added right at the start of incubation. For different wells, mosunetuzumab (mosu), obinutuzumab (obi), or IgG as the control were provided. (**A**) An isolated B cell in a co-culture with mosunetuzumab followed for roughly 2 h. Images were taken at the same position in the appropriate well every 7 min, as indicated in white. (**B**,**C**) Images were taken every 7 min for 364 min in channels for red, blue, and transmitted light at the same time. An exemplary presentation (**B**) and an overview (**C**) of how red areas, representing the amount of dead cells, increased according to the time and the antibody used are shown. (**D**) Areas under the curves (AUCs) presented in (**C**) were determined and compared. The data are presented as mean ± standard error of mean (SEM) of 5 independent experiments, * *p* < 0.05 comparing the obi result with the isotype control.

In order to shed light on the reaction kinetics of mosunetuzumab, we performed live cell imaging of γδ T cell and Hoechst 33258 stained B cell co-cultures after adding mosunetuzumab, obinutuzumab, or an isotype control. For labeling of apoptotic and dead cells, Apotracker™ Green and 7-Aminoactinomycin D (7AAD) were added right at the start of incubation. Apoptotic cells were supposed to exhibit a green rim, while dead B cells turned from blue to red. Figure 3A shows a blue-colored B cell encountered by multiple unstained γδ T cells over time. Tight adjacent membranes of B and γδ T cells turned green (minutes 21, 28, and 56), and the B cell‘s color changed from blue to purple by increasing 7AAD staining from minute 56 onward. For a quantitative approach, the number of cells was increased four times, and the observation period was extended from roughly 2 h to 6 h. While the appearance of the slightly green-colored cell rim was hard to detect unequivocally throughout the whole collection of images taken, Apotracker™ Green was omitted. Because dead cells turned red due to 7AAD staining, the relative size of red areas in the same image section was quantified and plotted over time. A representative example of images is shown in Figure 3B. Summarizing data from five independent experiments, the red areas (representing the relative amount of dead cells) appeared to increase faster due to mosunetuzumab vs. obinutuzumab or the isotype control, thereby reflecting an improved killing process (Figure 3C). For statistical analysis, we determined the area under the curves (AUCs) from 3C and showed the result in 3D. Only the difference between obinutuzumab and the control antibody was significant, whereby the lack of significance for mosunetuzumab was mainly due to the lack of normal distribution and the limited testing options for small samples. We definitely saw a trend that the AUC is higher for mosunetuzumab than for the control.

**Figure 4:**
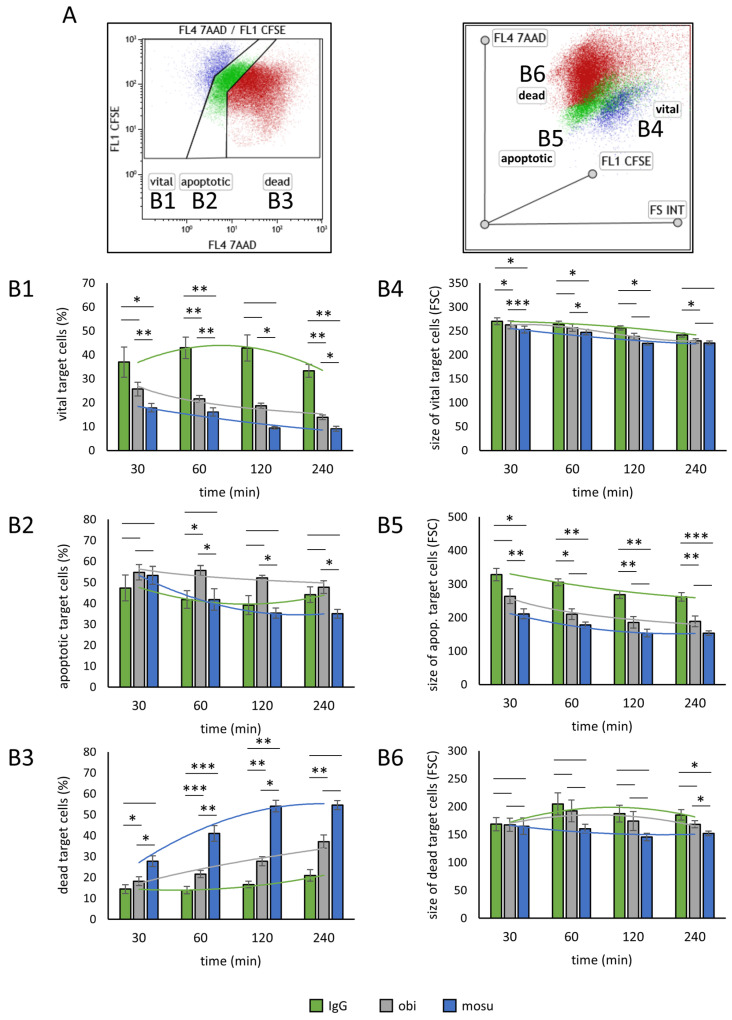
Flow cytometric analysis of B cell apoptosis driven by yδ T cells and therapeutic antibodies over time. B cell lymphoma cells were stained with CFSE and incubated with stimulated yδ T cells (>90% purity) in a 1:2 ratio. Therapeutic antibodies mosunetuzumab (mosu), obinutuzumab (obi), or IgG as the control were added. After indicated times, co-cultures were provided with 7AAD and analyzed through flow cytometry for relative amounts of vital (marked as “(**B1**)”), dead “(**B3**)”, or apoptotic “(**B2**)” target B cells, as shown in principle in (**A**). Changes in subpopulation fractions in relation to the time and the antibody used are shown in the corresponding Figures (**B1**–**B3**). As shown in the radar plot in (**A**), various FSC values for different subpopulations (here marked as (**B4**–**B6**)) can be observed. For a more precise approach, FSC values of the subpopulations are shown in relation to the time and the antibody used in the corresponding Figures (**B4**–**B6**). The summarized results of five independent experiments are presented as mean ± SEM, * *p* < 0.05, ** *p* < 0.01, *** *p* < 0.001.

Flow cytometry experiments were set up to complement observations from the microscopic analysis. Mosunetuzumab, obinutuzumab, or an isotype control were added to co-cultures of γδ T and B cells. Samples were analyzed after 30, 60, 120, and 240 min through flow cytometry. B cells were stained beforehand with carboxy–fluorescein succinimidyl ester (CFSE) for easy discrimination. As shown illustratively in Figure 4A, the intensity of 7AAD staining was used as a criterion for discriminating vital (4B1), apoptotic (4B2), and dead (4B3) target cells, as it has been done before [34]. Figure 4(B1) shows the severe reduction of vital cells by mosunetuzumab or obinutuzumab compared to the control antibody. With regard to dead cells, mosunetuzumab showed a steep ascent over time, while obinutuzumab revealed just a moderate increase compared to the effect of the control (Figure 4(B3)). Because obinutuzumab led to a substantial decrease in vital cells but not a marked increase in dead cells at the same time, the so-far-unassigned cells can be found in an apoptotic state (Figure 4(B2)). For a closer look at the data, we analyzed the forward scatter (FSC) values as a marker for the size of the different cell groups (Figure 4A, radar plot on the right side). While these values do not substantially differ between differently treated target cells within vital (Figure 4(B4)) or dead cells (Figure 4(B6)), there is a significant difference between the sizes of apoptotic target cells. Incubation with obinutuzumab or mosunetuzumab resulted in significantly smaller cells compared to incubation with the isotype control (Figure 4(B5)). In summary, mosunetuzumab induced a fast transition from “vital” to “dead” without target cells remaining in the apoptotic state. Obinutuzumab, on the contrary, mainly induced the transition of target cells from “vital” to “apoptotic” but not from “apoptotic” to “dead”. In conclusion, mosunetuzumab provided an improved killing process.

## 3. Discussion

γδ T cells appear to be powerful weapons against cancer cells due to several characteristics. Aside from their capacity to kill tumor cells, they also reveal tropism for many tissues and diminish the possibility for a graft-versus-host reaction in an allogeneic setting. γδ T cells have already been used for cancer treatment in different scenarios, including a combination with the mono-specific antibody obinutuzumab. On the other hand, mosunetuzumab is a bi-specific anti-CD3/anti-CD20 antibody that provides close proximity between conventional and/or γδ T and target B cells in general, and should increase cytotoxic activity [35]. In this study, we aimed to elucidate different aspects of the killing efficiency and cell–cell interaction dynamics with regard to the combination of γδ T cells and mosunetuzumab. Even in the absence of B cells, mosunetuzumab induced an increased expression of CD107a and IFN-γ by γδ T cells. It is quite likely that the cells were stimulated via anti-CD3/CD3 binding, as described by others [36]. Upon incubation with mosunetuzumab, γδ T cells showed enhanced killing activity towards B cells from B cell lymphoma cell lines, CLL patients, and healthy donors, as well. Therefore, the binding of CD3 and/or the physical proximity between γδ T cells and B cells, and not the recognition of specific malignant cell markers, are likely the critical factors for cell lysis in our experimental setting.

Obinutuzumab was shown to trigger a stronger ADCC effect of γδ T cells against lymphoma than rituximab, ofatumumab, or daratumumab [25,37], and it was subsequently used in our clinic in combination with γδ T cells to treat a patient suffering from Richter transformation who was not eligible for other options [18]. Therefore, we set up a comparative analysis of the cytotoxic effects of γδ T cells on B cells of different kinds when mosunetuzumab vs. obinutuzumab or an isotype control was administered. With regard to the four B cell lymphoma cell lines tested, mosunetuzumab achieved significantly better lysis than obinutuzumab for Granta only. In accordance with this, a former study by Young et al. showed Granta to be more susceptible than Daudi to the anti-CD20 antibody rituximab. Granta cells proliferated less and produced lower amounts of the pro-inflammatory cytokines IFN-γ, IL-2, and TNF-α and higher amounts of the anti-inflammatory cytokine IL-10, which may attenuate the cytotoxic T cell activity [38]. Additionally, in our own previous work, we observed a lower ADCC enhancing effect of obinutuzumab against Granta compared to other B cell lymphoma cell lines [25]. Furthermore, CD19- or CD37CAR T cells exhibited lower anti-lymphoma activity on Granta vs. U2932, Daudi, or Mino [39]. It is up to further studies to elucidate whether the reason for Granta’s characteristics may be found in the different origins of the lymphoma cell lines analyzed here.

Because it is widely accepted that cell lines and primary cells differ in many aspects [40], we included freshly malignant cells from CLL patients in our analyses. Therein, significantly higher specific lysis with mosunetuzumab was revealed, regardless of the effector-to-target ratio. Moreover, specific lysis with obinutuzumab incubation showed high standard deviations and presumably therefore, no significant difference in comparison to the control, even though significant results in similar experimental settings have been demonstrated in a former study by our lab [25]. However, the cultivation process of γδ T cells has changed between the former and the present study, thereby increasing the relative amount of γδ T cells from >70% to >90% and diminishing possible influences by natural killer (NK) cells and αβ T cells. In addition, the variabilities of target cells from different patients and effector cells from different healthy donors may prevent a congruent result, which is overcome with the application of mosunetuzumab. Similarly, past [25] and present research has not found a correlation between the expression levels of CD20 and lytic activity. This held true even though a clinical investigation correlated CD20 expression levels and infusion-related reactions in patients with CLL treated with obinutuzumab [41] before. In the context of lysis by the complement system, CD20 levels were found to determine the in vitro susceptibility to rituximab [42], the “parent compound“ of obinutuzumab.

In summary, mosunetuzumab initiated a significantly stronger cytotoxic effect on primary CLL cells than obinutuzumab. On B cell lymphoma cell lines, however, that difference could not be carved out that clearly. We hypothesized that the cytotoxic effect of both therapeutic antibodies on B cell lymphoma cell lines may have reached its utmost potential after 4 h of incubation time, resulting in no significant difference. Presumably, efficient varieties were therefore found after shorter incubation periods. For a closer look at the kinetics of the cytotoxic effects exerted by mosunetuzumab vs. obinutuzumab, we used live cell imaging to observe cell–cell contacts between γδ T and B cells. Additionally, we performed flow cytometric analysis over consecutive time periods. We could trace early and later formations of apoptotic and dead cell subpopulations. The transition of a vital cell into the apoptotic state or from there to death is obviously carried out at different speeds by mosunetuzumab vs. obinutuzumab.

Even though it may not be of central interest from a clinical perspective, it is tempting to speculate whether the modes of action for both therapeutic antibodies are substantially different. As mentioned above, obinutuzumab and mosunetuzumab bind to CD20 at partly overlapping epitopes but in different orientations. Furthermore, obinutuzumab provides two instead of one CD20 binding sites per antibody. Therefore, obinutuzumab not only binds to CD20 with a higher affinity than mosunetuzumab [10] but also generates CD20 pairs bound to one antibody molecule, resulting in different CD20 complex association and membrane compartmentalization [5]. Subsequently, obinutuzumab may induce apoptosis to a higher degree from the start [3,43]. After opsonization, immune cells (and, additionally, in an in vivo situation, parts of the complement system) are recruited to the malignant B cell by offering the Fc region as a binding site for distinct receptors on monocytes, macrophages, granulocytes, and NK or γδ T cells. Through glycoengineering, oligosaccharide residues in the Fc region of obinutuzumab have undergone defucosylation, which resulted in a higher affinity for FcγRIIIa (CD16a) and an increased activation of the bound immune cells [44,45,46]. Due to an amino acid exchange at position N297 in mosunetuzumab, glycosylation at that position is reduced, which leads to decreased binding activity by Fc-γ-receptors of granulocytes and NK cells [47]. Consequently, complement-dependent cytotoxicity and ADCC were expected to be diminished [48]. Still, activation and cytolytic capabilities of NK cells were found to be increased in a phase I trial with mosunetuzumab. Subsequent analysis in vitro and in a mouse model confirmed these findings [49]. Thus, mosunetuzumab obviously exhibits conventional ADCC effects, as well.

While obinutuzumab may exhibit increased binding affinity to CD20 and recruitment of immune cells by its Fc region, mosunetuzumab reveals presumably faster and more specific recruitment of (γδ) T cells through specific binding and activation of CD3^+^ cells. While the main mode of action is expected to be the physical-proximity-induced immune synapse with B cells, future studies have to elucidate to what extent, exactly, this process contributes to the overall reaction against B cells. This task is further complicated because CD3, as an epitope for mosunetuzumab, cannot be omitted from experimental settings. Alternatively, CD3-induced signal transduction by ZAP70 or Lck [50] could be diminished using RNA interference [51] or distinct inhibitors [52]. Therefore, mosunetuzumab would generate close proximity between T and B cells without stimulating the T cells. For a second control, mosunetuzumab could be replaced by a mono-specific CD3 antibody so that T cells would be activated without enforced subsequent binding to B cells via anti-CD20/CD20. Thirdly, Fab fragments of mosunetuzumab would still generate enforced physical contact between γδ T and B cells, thereby excluding unwanted conventional ADCC effects by immune cells expressing receptors for the Fc region. A comparison of the cytotoxic effects towards B cells by γδ T cells treated with mosunetuzumab or the mentioned controls could identify the proportion of the impact of the enforced physical proximity between T and B cells on the cytotoxic effect.

From the clinical perspective, however, the question arises regarding which possible side effects can be expected (and still be tolerated) from a highly effective combination therapy of γδ T cells and mosunetuzumab. Obinutuzumab and, in particular, mosunetuzumab both contribute to substantial depletion not only of malignant but also of healthy B cells. Of note, the killing effect on healthy B cells by obinutuzumab was published before [53]. As CD20 is expressed by all B cells, this finding is not surprising. The fact that healthy B cells are affected by therapy with CD20 antibodies is a common and manageable side effect. From a clinical perspective, this can be accepted, as the healthy B cells usually recover after therapy [54]. The much stronger effect of mosunetuzumab in combination with γδ T cells on healthy and malignant B cells is still advantageous if the much higher ratio of malignant vs. healthy B cells in a CLL patient’s blood is taken into account. Thereby, the probability of encountering and killing a malignant vs. a healthy B cell using a combination of mosunetuzumab and γδ T cells is much higher until both B cell subpopulations reach an equilibrium. Only comparative clinical studies can prove if the killing of healthy B cells limits broad administration or is still deemed tolerable. Another well-known side effect of bi-specific antibodies, such as mosunetuzumab, is the development of cytokine release syndrome [55]. As this is also observed for cellular therapies, like CAR T cells [56], it is possible that a combined therapy of bi-specific antibodies and stimulated γδ T cells can increase the risk, even with appropriate pre-treatment of the patients. Overall, thoroughly designed clinical studies are needed to prove the safety of a combination therapy of stimulated γδ T cells and bi-specific antibodies.

Taken together, the bi-specific anti-CD3/anti-CD20 antibody mosunetuzumab reveals a much stronger cytotoxic effect by γδ T cells on B cells from B cell lymphoma cell lines, healthy donors, and CLL patients than the mono-specific anti-CD20 antibody obinutuzumab. This may be partly due to the close proximity of target and effector cells, an improved killing process, and/or the increased activation of T cells with regard to CD107a and IFN-γ expression through CD3 stimulation. These results are in line with the expectation of superior therapeutic effects of bi-specific vs. mono-specific antibodies [57,58]. Future studies could elucidate possible effects of other bi-specific anti-CD3/anti-CD20 antibodies, such as epcoritamab, glofitamab, or odronextamab. Furthermore, tri-specific antibodies are in the pipeline for (pre-)clinical studies [59], including the anti-CD3/anti-CD20/anti-CD19 antibody “A2019”, which was already characterized in comparison to blinatumomab by Wang et al. [60]. With regard to the preclinical analyses presented here, the combination of γδ T cells and mosunetuzumab was deemed to be a promising therapeutic option for the treatment of hematological cancers. Future clinical studies are needed to prove the safety and efficacy of this concept in vivo.

## 4. Materials and Methods

### 4.1. Cell Culture, Cell Isolation, and Ex Vivo Stimulation of γδ T Cells

Our investigations were performed with peripheral blood obtained from healthy adult donors or CLL patients. The studies involving human participants were reviewed and approved by the ethics committee of the Bavarian State Medical Association (EC number: 23053). Written informed consent to participate in this study was provided by the participants. All donors signed an agreement according to the General Data Protection Regulation.

#### 4.1.1. Isolation and Stimulation of γδ T Cells

MNCs from healthy donors were isolated through density gradient centrifugation with Biocoll (Bio&SELL, Feucht, Germany). For cultivation according to the Ko–Op protocol [32], MNCs at a concentration of 5E5/mL were cultured in 50 mL cell culture flasks (Sarstedt, Nuembrecht, Germany) using medium consisting of OpTmizer^TM^ CTS^TM^ T-Cell Expansion Basal Medium, OpTmizer^TM^ CTS^TM^ T-Cell Expansion Supplement (Gibco/Thermo Fisher, Waltham, MA, USA) and 1% 200 mM L-glutamine (Bio&SELL, Feucht, Germany). On day 0, 10 µM of zoledronate (Sigma-Aldrich, St. Louis, MO, USA) was added, and 1000 U/mL of interleukin-2 (IL-2) (Burton-on-Trent, UK) was added on day 2. On days 4, 7, and 9, half of the medium was removed and replaced with fresh medium containing 2× 1000 U/mL IL-2 for a final concentration of 1000 U/mL.

#### 4.1.2. Isolation and CD19 Selection of MNCs from Healthy Donors

MNCs from healthy donors were isolated through density gradient centrifugation with Biocoll (Bio&SELL, Feucht, Germany). CD19^+^ cells were selected using the MidiMACS system and the REAlease anti-CD19 MicroBeads (both from Miltenyi Biotec, Bergisch Gladbach, Germany) according to the manufacturer’s instructions, except human serum albumin (HSA) (CSL Behring GmbH, Marburg, Germany) was used instead of bovine serum albumin (BSA) as part of the washing buffer. The CD19-depleted MNCs were used for experiments on the same day. The MicroBeads were removed from the CD19^+^-selected cells using the REAlease CD19 MicroBead Kit (Miltenyi Biotec, Bergisch Gladbach, Germany) and stored overnight in PBS in the refrigerator before being used for experiments.

#### 4.1.3. Isolation and CD3 Depletion of Patient Samples

MNCs from CLL patients were isolated through density gradient centrifugation with Biocoll (Bio&SELL, Feucht, Germany). CD3^+^ cells were depleted using the MidiMACS system and anti-CD3 MicroBeads (both from Miltenyi Biotec, Bergisch Gladbach, Germany) according to manufacturer’s instructions, except HSA (CSL Behring GmbH, Marburg, Germany) was used instead of BSA as part of the washing buffer. In order to discriminate the different mononuclear cell populations and to confirm the success of depletion, the cells were stained before and after with anti-CD19-Fluorescein isothiocyanate (FITC), anti-CD3-r-phycoerythrin-Texas Red (ECD), anti-CD200- phycoerythrin-cyanine 7 (PC7), anti-CD5-Allophycocyanin (APC), and anti-CD45- Krome Orange (KrO). Afterwards, the cells were frozen and stored in liquid nitrogen. One day before the experiments, CD3^-^ MNCs were thawed and cultured in standard medium consisting of RPMI 1640 supplemented with 10% fetal bovine serum (FCS), 1% 200 mM L-glutamine, and 1% penicillin/streptomycin (all from Bio&SELL, Feucht, Germany) overnight. Cells were again stained with anti-CD19-FITC, anti-CD3-ECD, anti-CD200-PC7, anti-CD5-APC, and anti-CD45-KrO.

#### 4.1.4. Culture of Cell Lines

The B cell lymphoma cell lines Jeko, Mino, Daudi, U2932, and Granta were obtained from the German collection of microorganisms and cell cultures (DSMZ, Braunschweig, Germany) and cultured in standard medium consisting of RPMI 1640 supplemented with 10% fetal bovine serum (FCS), 1% 200 mM L-glutamine, and 1% penicillin/streptomycin (all from Bio&SELL, Feucht, Germany). Cell counts and cell viability were established using a hemocytometer and the trypan blue exclusion method.

### 4.2. Flow Cytometry and Antibodies

A Navios flow cytometer (Beckman Coulter, Brea, CA, USA) was used for multicolor immunofluorescence and functional tests. Cells were stained in appropriate combinations according to their application with the following antibodies: anti-CD3- ECD [clone UCHT1], anti-CD5- APC [clone CLB-T1/1], anti-CD19- FITC [clone J3-119], anti-CD45- KrO [clone J33], anti-CD56- PC7 [clone N901], and anti-CD200-PC7 [clone OX-104] (all from Beckman Coulter, Brea, CA, USA); anti-T-cell receptor (TCR) γδ-FITC [clone 11F2], anti-CD107a-phycoerythrin (PE) [clone H4A3], and anti-IFN-γ-PE [clone REA600] (all from Miltenyi Biotec, Bergisch Gladbach, Germany); and anti-CD20-Pacific Blue (PB) [clone 2H7] (BioLegend, San Diego, CA, USA). As negative controls, anti-IgG-PE [clone IS5-32F5] (Miltenyi Biotec, Bergisch Gladbach, Germany) and anti-IgG2b-PB [clone MPC-11] (BioLegend, San Diego, CA, USA) were used. γδ cells were defined as CD3^+^ TCRγδ^+^ MNCs, CLL cells as CD5^+^ CD19^+^ MNCs, B cells as CD19^+^ MNCs, and T cells as CD5^+^ MNCs. For functional assays, therapy-grade monoclonal antibodies obinutuzumab and the bi-specific antibody mosunetuzumab (both Roche, Grenzach-Wyhlen, Germany), as well as the IgG1-kappa control antibody (Sigma-Aldrich, St. Louis, MO, USA), were used.

### 4.3. Cytotoxicity Assay

Cytotoxicity experiments were conducted in 96-well V-bottom plates (Greiner bio-one, Frickenhausen, Germany) as co-cultures at different effector to target cell ratios with different concentrations of mosunetuzumab, 1 µg/mL of obinutuzumab, or 1 µg/mL of isotype control antibody. Different B cell lymphoma cell lines, B cells from healthy donors, and CD3-depleted MNCs from CLL patients were used as target cells and previously labeled with CFSE (BioLegend, San Diego, CA, USA). Stimulated MNCs from healthy donors were used as effector cells. Following co-culture of effector cells, target cells, and the indicated therapeutic antibody for 4 h, the cells were harvested and technical replicates were pooled, treated with 7AAD (BioLegend, San Diego, CA, USA), and analyzed through flow cytometry. Specific cell-mediated cytotoxicity was expressed as “specific lysis (%)” and calculated using the formula specific lysis (%) = [% 7AAD^+^ target cells in the respective effector to target ratio − % 7AAD^+^ target cells in target cell only culture] × 100/[100 − % 7AAD^+^ target cells in target cell only culture]. If indicated, the lytic units per 1E6 effector cells were calculated according to Bryant et al. based on specific lyses obtained from different effector to target ratios. A lytic unit is the number of effector cells required to lyse a specific percentage of target cells. To present the results using a value that increases with the level of cytotoxicity, the number of lytic units contained in 1E6 effector cells was calculated. The following formula was used: $lytic\;units\;per\;10^{6}\;effector\;cells=10^{6}\cdot exp\left(\frac{\bar{Y^{*}}-Y_{p}^{*}}{C}\right)/(T\cdot \bar{X_{G}}$) [61]. *Y* is the specific lysis measured in a defined effector-to-target ratio. *Y* is logistically transformed (Y*) using the following formula: $Y^{*}=LN(\frac{Y}{100-Y})$. We defined a reference lysis p of 60%, which is also logistically transformed ($Y_{p}^{*}).$ $\bar{Y^{*}}$ is the arithmetic mean of the logistically transformed specific lyses measured in each effector to target ratio, C is a constant with relation to the slope of the curve (defined as 1), T is the number of target cells $\left(10^{4}\right)$, and $\bar{X_{G}}$ is the geometric mean of the effector to target ratios used in the assay.

### 4.4. Degranulation Assay

As a marker of degranulation, the surface antigen CD107a was examined in our degranulation assay. For this, stimulated MNCs were co-cultured with Jeko in a 1:2 ratio or with culture medium with the addition of mosunetuzumab in respective concentrations or 1 µg/mL of isotype control and the fluorescent antibody anti-CD107a-PE or anti-IgG-PE as the control in 96-well V-bottom plates (Greiner bio-one, Frickenhausen, Germany) for 3 h. Subsequently, the cells were washed, the surface antigens were stained with anti-TCR γδ-FITC and anti-CD3-ECD, and the cells were analyzed through flow cytometry. To define CD107a^+^ γδ T cells, cells were stained with isotype control, and the gate was adjusted so that 2% of the cells in the isotype control were defined as positive. The ΔMFI of CD107a was calculated as MFI (CD107a) minus MFI (isotype).

### 4.5. IFN-γ and CD107a Assay

For the measurement of IFN-γ and CD107a intracellular production in response to target cells, 1E6 freshly isolated, unstimulated MNCs and 6E5 Jeko cells were co-cultured in a final volume of 1 mL per well in a 48-well plate (Greiner bio-one, Frickenhausen, Germany) in standard medium. If indicated, MNCs were depleted for B cells, as described above. After the addition of mosunetuzumab in the respective concentrations or 1 µg/mL of isotype control, the cells were incubated for 48 h. Then, 300 µL of the supernatant was removed, and anti-CD107a-FITC was added and incubated with the cells for another 5 h. After staining the surface antigens with anti-CD3-ECD and anti-TCR γδ-PC5, intracellular IFN-γ was stained with anti-IFN-γ-PE using the inside stain kit (Miltenyi Biotec) according to the manufacturer’s instructions.

### 4.6. Live Cell Imaging

B cells from the lymphoma cell line Mino were stained with Hoechst 33258 (Thermo Fisher, 1:10,000 in PBS) for 5 min in the dark at room temperature and subsequently sorted for appropriate FSC/side scatter (SSC) values and successful staining as criteria in a cell-sorting device (Cytoflex SRT from Beckman Coulter, Brea, CA, USA). Cells were stored at 4 °C overnight and then used for co-culture assays with unstained stimulated γδ T cells after adding Apotracker™ Green and 7AAD (both from BioLegend, San Diego, CA, USA). Co-cultures were set up in different wells of µ-Slide 8 Wells (ibidi, Gräfelfing, Germany) with IgG1-kappa control antibody (Sigma-Aldrich, St. Louis, MO, USA), obinutuzumab, or mosunetuzumab (both Roche, Grenzach-Wyhlen, Germany) at 1 µg/mL. Cell counts differed according to the intended purpose: 5E4 B cells + 1E5 γδ T cells for documenting individual cell–cell interactions and 2E5 B cells + 4E5 γδ T cells for enabling and observing a steady apoptotic process over time. Co-culture at 37 °C and 5% CO_2_ and documentation through life cell imaging were carried out using a suitable fluorescence microscope (BZ-X810 from Keyence, Osaka, Japan) for up to 6 h. Images were captured at the same position in the appropriate well every 7 min for green, red, and blue fluorescence and transmitted light. To analyze the apoptotic process over time, images displaying red fluorescence were analyzed for the relative amount of red areas on each image using the software Image Color Summarizer v0.80 (Leizersoft, Yoqne‘am ‘Illit, Israel). Percentages of red areas were plotted over time, and the areas under the distinct curves (AUCs) were calculated.

### 4.7. Flow Cytometric Analysis of B Cell Apoptosis

B cell lymphoma cells were initially labeled with CFSE (BioLegend, San Diego, CA, USA) and then incubated with γδ T cells in capped FACS tubes in a 1:2 ratio at 37 °C and 5% CO_2_. Therapeutic antibodies, such as mosunetuzumab, obinutuzumab, or IgG as the control, were added at a 0.5 µg/mL final concentration. After 30, 60, 120, or 240 min, co-cultures were stained with 7AAD and analyzed through flow cytometry for relative amounts of vital, dead, or apoptotic target B cells, as shown in principle in former studies [34,62]. Additionally, FSC values of the described B cell subpopulations were determined.

### 4.8. Data and Statistical Analysis

Data were analyzed with the software Kaluza analysis V2.1 (Beckman Coulter, Brea, USA), Excel 2016 (Microsoft, Redmond, WA, USA) and SPSS Statistics 28 (IBM, Armonk, NY, USA). Data are presented as mean ± standard deviation (SD) or ± standard error of the mean (SEM) as indicated. The normal distribution of the data was verified using the Shapiro test. Levels of significance were calculated using the paired *t*-test or the Wilcoxon test. *p* < 0.05 is considered statistically significant.

## Data Availability

The datasets generated for this study are available upon request to the corresponding author.

## Abbreviations

The following abbreviations are used in this manuscript:

Abbreviation	Explanation
7AAD	7-Aminoactinomycin D
ADCC	antibody-dependent cell cytotoxicity
BSA	bovine serum albumin
CAR T cells	chimeric antigen receptor T cells
CFSE	carboxy–fluorescein succinimidyl ester
CLL	chronic lymphocytic leukemia
ECD	electron-coupled dye
EGFR	Epidermal Growth Factor Receptor
FasL	Fas-ligand
FITC	Fluorescein isothiocyanate
FSC	forward scatter
HSA	human serum albumin
IFN-y	interferon-γ
IL-2	interleukin-2
KrO	Krome Orange
MFI	mean fluorescence intensity
MHC	major histocompatibility complex
MNCs	mononuclear cells
mosu	mosunetuzumab
NK cells	natural killer cells
obi	obinutuzumab
PB	Pacific Blue
PC5	phycoerythrin-cyanine5
PC7	phycoerythrin-cyanine7
PD-L1	programmed cell death ligand 1
PE	phycoerythrin
SD	standard deviation
SEM	standard error of mean
SSC	side scatter
TCR	T cell receptor
TNF	tumor necrosis factor
TRAIL	TNF-related apoptosis-inducing ligand
Zol	zoledronate
